# Impact of *lian gong* on the quality of life of individuals with dizziness in primary care

**DOI:** 10.11606/s1518-8787.2019053001234

**Published:** 2019-09-12

**Authors:** Aline Lamas Lopes, Stela Maris Aguiar Lemos, Pedro Henrique Scheidt Figueiredo, Juliana Nunes Santos

**Affiliations:** I Universidade Federal de Minas Gerais, Faculdade de Medicina, Programa de Pós-Graduação em Ciências Fonoaudiológicas. Belo Horizonte, MG, Brasil; II Universidade Federal de Minas Gerais, Faculdade de Medicina, Departamento de Fonoaudiologia. Belo Horizonte, MG, Brasil; III Universidade Federal dos Vales do Jequitinhonha e Mucuri (UFVJM), Faculdade de Ciências Biológicas e da Saúde, Departamento de Fisioterapia. Diamantina, MG, Brasil

**Keywords:** Dizziness, rehabilitation, Complementary Therapies, Primary Health Care, Randomized Controlled Trial

## Abstract

**OBJECTIVE:**

To assess the effects of the *lian gong* practice as a rehabilitation strategy in primary health care on the quality of life and functional capacity of people with dizziness.

**METHODS:**

Randomized controlled clinical trial. Thirty-six people, who were complaining of dizziness or vertigo without the presence of central signs and were referred by the physician of primary health care participated in the study. The individuals were randomly allocated to the three experimental conditions: *lian gong* group (n = 11), vestibular rehabilitation group (n = 11) and control group (n = 14). The interventions were weekly, in group, with duration of 12 sessions. The participants were evaluated before and after the intervention regarding quality of life by the 36-Item Short Form Health Survey and the functional capacity by the Short Physical Performance Battery.

**RESULTS:**

The scores of all domains of the Short Form Health Survey increased after intervention in the *lian gong* group. This variation was higher than that observed in the control group for the domains functional capacity, limitation by physical aspects and general health status, and also higher than that found after the intervention in the Vestibular Rehabilitation Group regarding pain. No differences were found in *t*he Short Physical Performance Battery.

**CONCLUSIONS:**

Based on the results presented, *lian gong* improves the quality of life of individuals with dizziness, without altering the functional capacity.

## INTRODUCTION

Studies reveal the growing use of complementary and alternative medicine (CAM) worldwide as a way to improve health and welfare, as well as to relieve symptoms associated with chronic diseases or side effects of conventional treatments^1,2^. The interest in the use of CAM increased mainly in developed countries such as France, Canada, Germany and Italy, where 70% to 90% of the population use their resources and therapeutic practices^1^. In the United States, we observed in 2012 that 33% of adults used some complementary health practice^2^.

In Brazil, complementary therapies are called integrative and complementary practices (ICP). They were instituted by ordinance 971/2006 of the Ministry of Health^3^ and correspond to a set of therapies that include oriental physical exercises such as *lian gong* (LG), tai chi chuan, and acupuncture, auriculotherapy, homeopathy, termalism, phytotherapy and oriental massage. The use of alternative and complementary therapies is on the rise, as well as its acceptance in the control and treatment of various chronic health conditions, such as HIV/AIDS, hypertension, high cholesterol, insomnia, bronchitis, diabetes, cancer^1^, dizziness and vertigo^4^, among others.

Dizziness is among the most common reasons to seek a medical appointment in primary health care (PHC). It is considered the most frequent symptom worldwide, occurring in all age groups, especially in adults and older adults^5^, being multifactorial in most cases, of vestibular and non-vestibular origin^6^. Among the causes of vestibular origin stand out the benign paroxysmal positional vertigo, presbyvertigo, ménière’s disease, vestibular neuritis, vertebrobasilar insufficiency, vestibular migraines and vestibulopathies secondary to labyrinthine infections^6^. The several non-vestibular causes are the diseases that directly alter these functions, especially the cardiovascular and orthopedic ones and decreased visual acuity^6,7^.

We believe that dizziness and vertigo, as symptoms of vestibular disorders, are present in 5% to 10% of the world population, being the most common symptom after 65 years^6,7^. One third of older adults are likely to have symptoms of dizziness in the period of one year^6^.

The literature presents as forms of treatment of dizziness and vertigo the use of medication, surgical resources, traditional vestibular rehabilitation (VR) and VR with the use of technological innovations and virtual realities, besides the complementary alternative practices^4^. Evidence shows the positive effects of VR on PHC, with improvements in postural control, functional capacity and quality of life of patients, and this therapeutic option is the most approached in the rehabilitation of individuals^8^. Among the alternative practices, the acupuncture and Tai Chi therapies have shown favorable results, indicating improvement of the balance and quality of sleep, as well as reduction of the risk of falls in individuals with dizziness^4,10,11^.

In Brazil, LG has been performed in PHC as part of the National Policy of Integrative and Complementary Practices in SUS^3^. LG is a therapeutic gymnastics consisting of exercises based on knowledge and experience of the Chinese body and martial arts. It stimulates the persistence of training and exercising the body by firm and gentle movements that minimize and eliminate muscular tensions, lengthen ligaments and tendons, correct the physical posture, stimulate the perception and integration of the senses and optimize motor coordination, balance and body consciousness, in addition to promoting the harmonization between body and mind, reducing thus the symptoms of anxiety and depression^12,13^.

The complete system of LG consists of three parts, totaling 54 exercises. The first part (anterior) comprises 18 movements to prevent and treat pain in the neck, shoulders, back, lumbar region, gluteus and legs. The second part (posterior), also consisting of 18 exercises, is intended for the prevention and treatment of joint pain, tenosynovitis and dysfunctions of the internal organs. The third part, called *i qi gong*, includes a set of 18 exercises aimed at the prevention and treatment of chronic bronchitis and the functional weakness of the heart and lungs, as well as other chronic airway diseases. The performance of the exercises is accompanied by music played by Chinese instruments, whose arrangement establishes the appropriate rhythm for the execution of the movements. Thus, each part of 18 exercises takes approximately 12 minutes to be held^12,13^.

In all parts, cephalic rotation movements are performed associated with eye movements of persecution and visual fixation, simultaneous or not to broader bodily movements, which stimulate the vestibulo-ocular and vestibulospinal systems, essential in the process of vestibular rehabilitation^4^. In both the anterior and posterior parts of the LG, there is lateral or anterosuperior cephalic movement, and the fixation and eye pursuit movements can be observed in 100% of the exercises. The practice of the *i qi gong*, in addition to requiring cephalic movement and movements of persecution and ocular fixation, exercises the static and dynamic balance of individuals with bodily exercises alternating open and closed eyes^12,13^. As in VR, it is believed that the repetition of the exercises practiced in the LG promotes visual stabilization and increases the vestibule-visual interaction during head movement, providing better static and dynamic stability in situations of sensory conflict, which enhances the mechanisms of vestibular adaptation^4,6,14^.

No studies investigating the effects of this complementary integrative practice were found on individuals with dizziness. However, it is believed that the LG, due to its peculiar characteristics, is beneficial to this population and reduces the impact of dizziness on the quality of life of the participants. Also, it is noteworthy, as advocated by the World Health Organization, the emerging need for scientific research to assess the quality, safety and effectiveness of CAM^1^ practices around the world.

Thus, the aim of this study is to evaluate the effects of the LG practice as a rehabilitation strategy in PHC on the quality of life and functional capacity of people with dizziness.

## METHODS

### Design

This is a randomized controlled clinical trial with two-arm parallel design. The project titled “The Feasibility and Effectiveness of the Vestibular Rehabilitation Program in Primary Health Care” was approved by the Institutional Research Ethics Committee of the Universidade Federal de Minas Gerais under the number CAAE 15987713.5.00005149 and by Brazilian Registry of Clinical Trials under the code RBR-2nxt6y. All participants signed the informed consent form.

The managers of the participating health units were informed about the accomplishment of the study and signed the consent letter. PHC physicians who voluntarily agreed to participate in the research referred patients with complaints of dizziness or vertigo without the presence of signs and symptoms of central alterations through the matrix of the family health team (FHS) with the family health support nucleus (NASF).

### Study Location

This study was conducted in two health centers of SUS in a Brazilian metropolis.

### Sample

The individuals were recruited to participate in the study from May to December 2016. The inclusion criteria were: age 18 years or older, being a SUS patient and resident or worker in the areas covered by the health centers participating in the study, having complaints of dizziness or vertigo with absence of signs or central symptoms, medical indication for participation in the proposed groups and signing of the informed consent form. Exclusion criteria were: presence of intellectual or physical disability or mental disorders that prevented the accomplishment of the activities proposed in the groups, withdrawal or non-adherence to treatment with more than four absences to the meetings, pregnancy and presence of speech-language pathology assessment compatible with benign paroxysmal positional vertigo (BPPV).

To determine the number of subjects, the statistical program G*Power 3.1 was used. The sample size was based on the study by Yardley et al.^17^, with the results of the comparison of the mean scores and standard deviation (SD) of the Dizziness Handicap Inventory (DHI) between two groups of patients participating in a randomized clinical trial to verify the effectiveness of vestibular rehabilitation in PHC. For this trial, nine users were required for each group, considering power (Beta type I error) of 95%, alpha equal to 0.05 and effect size of 1.95. Losses increased by 20% (n = 5), totaling 32 subjects.

The subjects were referred by the PHC physician and evaluated by a veiled researcher for the groups. After analyzing the inclusion and exclusion criteria, the subjects were randomly allocated into three groups: LG group, complementary/integrative method; VR group, conventional method, and control group (CG). Randomization was performed using a simple draw. Paper strips containing the indication of one of the three groups were allocated in opaque envelopes. At the time of the entry of the volunteer in the study, a researcher drew the group in which the volunteer would participate.

To characterize the sample, instruments elaborated by the researchers were used to evaluate the socio-demographic aspects, the balance and the complaints, signs and symptoms of dizziness and vertigo of the volunteers. For the latter, information was collected about the patients’ clinical history and their relation with the complaint made.

### Interventions

The proposed intervention for the LG group was based on the protocol recommended by the author of the technique, Dr. Zuang Yuan Ming, according to the class model already established by the city of Belo Horizonte: anterior series, posterior series and *i qi gong.* Fifty-four exercises were performed, coordinated with breathing, in a slow and continuous manner, which act in the individual as a whole. In addition to treating and preventing musculoskeletal pain, these exercises optimize cardiorespiratory function, stimulate balance mastery, body consciousness, postural stabilization and ocular fixation ^12,13,18^. In the VR group, the intervention was based on the protocols for vestibular rehabilitation of Norré^14^, Cawthorne and Cooksey^15^ and Herdman^16^, with the selection of exercises For postural stabilization, ocular fixation and training to maintain balance. The individuals in the control group did not receive treatment.

The interventions (VR and LG) were held by one of the researchers, a speech therapist in the NASF. The sessions of 50 minutes were held weekly on a collective basis (groups between five and seven participants). The interventions began in July 2016 and ended in April 2017, so that the patients referred were inserted in the groups shortly after the initial evaluation and remained until completing 12 sessions.

### Outcomes

Pre- and post-intervention evaluation were performed by a speech therapist who did not know to which group the patient belonged. The primary outcomes were the results of the generic questionnaire for quality of life assessment 36-Item Short Form Health survey (SF36)^19^. The functional capacity of the participants, assessed by the Short Physical Performance Battery (SPPB)^20^, was considered as a secondary outcome.

The SF36 was created to be a generic assessment questionnaire in two parts: the first to assess the health status (with questions related to physical mobility, pain, sleep, energy, social isolation and emotional reactions) and the second part to assess the impact of the disease on the patient’s daily quality of life. This is a multidimensional questionnaire consisting of 36 items, subdivided into eight scales or components: functional capacity, physical aspects, pain, general health status, vitality, social aspects, emotional aspects and mental health. The SF36 was analyzed by the final score ranging from zero to one hundred points (obtained by calculating the raw scale), in which zero reflects the worst health status and one hundred, the best^19^.

The Short Physical Performance Battery (SPPB) was adapted to the Portuguese language by Nakano^20^. It is a widely used test in clinical practice for functional evaluation of older adults. It comprises three stages, each with a score of up to four points, totaling the final score of at most 12 points. In the first stage, the individual remains initially in orthostatic position with the feet in parallel, then with the feet in semi-tandem and then in tandem stance. In the second stage, the gait velocity was measured in a four-meter course, dividing the distance traveled by the time spent. In the third stage, the time the older adult takes to perform the chair stand test for five times was measured^20^. The total score of the SPPB was the sum of the results of the balance tests, gait velocity and lower limb strength. The individuals who obtained between zero and six points were classified as low performance, between seven and nine points, intermediate performance, and between 10 and 12, high performance.

### Statistical Analysis

Statistical analysis was performed using the software SPSS 19.0 (Statistical Package for the Social Sciences). The Shapiro-Wilk test was used to analyze the normal distribution of continuous variables, and the data are presented as mean and standard deviation. The sample characterization variables were compared between the groups by unidirectional variance analysis (one way) or Kruskal-Wallis test, according to the normality test, with Tuckey *post hoc* test, if necessary. The comparison of the distribution of categorical variables between the groups was performed by the Chi-square test and is presented in absolute numbers and relative frequencies.

The effects of interventions in the SF36 domains were compared through confidence intervals of 95% (95%CI) of the differences between the averages at the initial and final moments, as well as the intergroup differences (LG *versus* VR; LG *versus* GC; VR *versus* CG) in the initial and final moments. The comparison between the differences was performed by one-way ANOVA with Tuckey *post hoc* test. The chi-square test was used to analyze the results of SPPB. A 5% significance level was adopted.

## RESULTS

**Figure 1:**
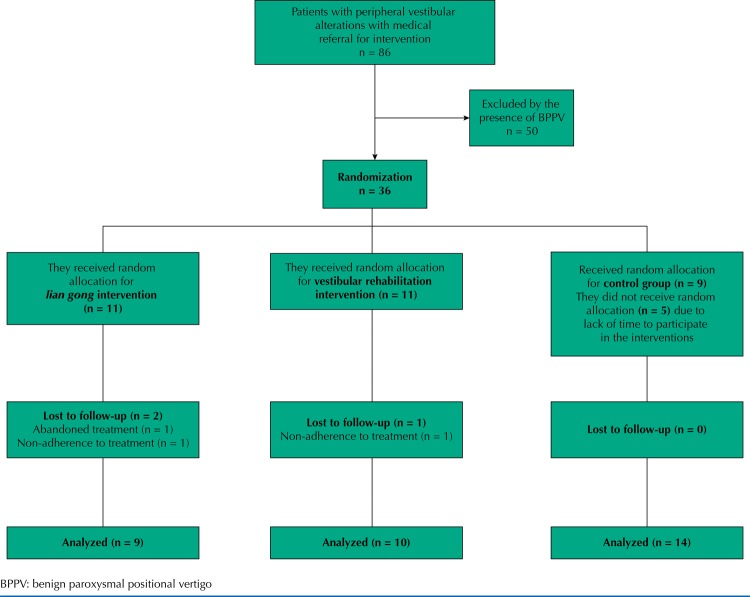
Flowchart of participation in the randomized clinical trial.

Figure 1 shows the flow of volunteers in the study. Initially, 86 patients were referred for possible participation in the research, but 50 were excluded because they presented clinical signs compatible with BPPV during the speech-language pathology assessment. Thus, 36 volunteers were randomly allocated to the three experimental conditions, 11 (31%) were in the LG group, 11 (31%) in the VR group and 14 (38%) in the CG. After the interventions began, an individual from the LG group abandoned treatment due to a job opportunity at the same time and two missed more than four sessions, one from the LG group and one from the VR group. Thus, 33 users who completed the study were analyzed, 29 (87.9%) women, with a mean age of 63 years (SD = 5.17), a minimum of 52 and a maximum of 72 years (Table 1).

BPPV: benign paroxysmal positional vertigo

**Table 1:** Characterization of the participants in the study.

Variable	Intervention (n = 9)	Intervention (n = 10)	Control (n = 14)	p
		
	(LG)	(VR)	(CG)	
		
	Mean and standard deviation/n and relative freq. (%)	Mean and standard deviation/n and relative freq. (%)	Mean and standard deviation/n and relative freq. (%)	
Sociodemographic aspects				
Age^a^ (years)	64 (4.5)	64.7 (4.4)	61.1 (5.6)	0.20
Women^b^	8 (88.8)	9 (90)	12 (85.7)	0.94
Body function and structure				
Dizziness^b^	9 (100)	10 (100)	14 (100)	1.00
Vertigo^b^	9 (100)	10 (100)	14 (100)	1.00
Anxiety^b^	9 (100)	8 (80)	11 (78.5)	0.33
Buzz^b^	6 (66.6)	7 (70)	11 (78.5)	0.80
Hollow head sensation^b^	7 (77.7)	5 (50)	6 (42.9)	0.24
Blurred vision sensation^b^	6 (66.5)	4 (40)	6 (42.9)	0.43
Visual changes^b^	7 (77.7)	9 (80)	12 (85.7)	0.75
Limitation of activities				
To stumble	3 (33.3)	4 (40)	9 (64.3)	0.28
To stagger	5 (55.5)	9 (90)	12 (85.7)	0.13
To turn the foot	4 (44.4)	3 (30)	10 (71.4)	0.12
To fall	3 (33.3)	5 (50)	10 (71.4)	0.19
To not perform physical activity	5 (55.5)	6 (60)	10 (71.4)	0.71
Other health conditions				
SAH	5 (55.5)	5 (50)	4 (28.6)	0.37
Diabetes	3 (33.3)	1 (10)	3 (21.4)	0.46
Depression	3 (33.3)	3 (30)	2 (14.3)	0.51
Migraine	2 (22.2)	5 (50)	2 (14.3)	0.14

LG: *lian gong*; VR: vestibular rehabilitation; CG: control group; Freq.: frequency; SAH: systemic arterial hypertension

^a^ ANOVA

^b^ Chi-square test.

Table 1 shows the characteristics of the patients in the LG, VR and CG groups. The groups did not differ in relation to the demographic characteristics, body function and structure, limitation of activities and other health conditions (p > 0.05). Table 2 shows the intragroup and intergroup analyses of the quality of life of the patients at the initial and final moments.

**Table 2:** Effects of interventions on quality of life.

Domains of SF36	Groups			Intergroups difference		
	
	*Lian gong* (LG)	Vestibular rehabilitation (VR)	Control (CG)	LG *versus* CG	VR *versus* CG	LG *versus* VR
General health status						
Pre	81.4 (3.2)	81.7 (4.6)	80.5 (1.7)	0.94 (-1.1–3.0)	1. 2 (-1.5–3.9)	0.25 (4.2–3.6)
Post	84.8 (4.1)	83.1 (4.8)	80.5 (1.5)	4.3 (1.7–6.8)^a^	2.5 (-0.34–5.4)	1.7 (-2.5–6.1)
≠ intragroups	3,44 (0,7–6,1)^a,b,c^	1,4 (0,01–2,7)^a^	0,71 (-0,5–0,6)			
Functional capacity						
Pre	69.7 (7.3)	67.4 (6.3)	71.8 (7.1)	-2.0 (-8.5–4.3)	- 4.4 (-10.3–2.8)	2.3 (-4.2–8.9)
Post	72.8 (7.1)	69.2 (6.3)	71.6 (6.9)	0.91 (-5.3–7.1)	-2.4 (-8.1–3.3)	3.3 (-3.3–9.8)
≠ intragroups	2.77 (1.2–4.3)^a.b^	1.8 (-0.5–4.1)	-0.21 (-1.3–0.8)			
Limitation by physical aspects						
Pre	82.7 (4.2)	80.9 (4.2)	81. 0 (4.4)	1.8 (-2.1–5.7)	-0.1 (-3.8–3.6)	1.8 (-2.2–6.0)
Post	84.3 (4.5)	82.4 (5.2)	80.5 (4.8)	2.0 (-0.47–7.9)	1.8 (-2.5–6.1)	1.9 (-2.8–6.7)
≠ intragroups	1.5 (0.3–2.7)^a.b^	1.5 (0.2–2.7)^a.b^	-0.42 (-1.4–0.5)			
Pain						
Pre	78.4 (5.3)	79.0 (5.8)	81.2 (3.0)	-2.8 (-6.4–0.8)	-2.2 (-6.0–1.4)	-0.55 (-5.9–4.8)
Post	8.18 (4.8)	77.3 (8.3)	81.2 (2.9)	0.67 (-2.6–4.0)	-3.9 (-8.8–1.0)	4.5 (-2.1–11.2)
≠ intragroups	3.4 (0.8–6.0)^a.c^	-1.7 (-6.7–3.3)	-0.07 (-0.9–0.7)			
Vitality						
Pre	60.3 (4.1)	59.9 (3.2)	61.5 (2.8)	1.4 (-4.1–1.8)	-1.6 (-4.1–0.97)	0.43 (-3.1–4.0)
Post	63.3 (5.0)	62.7 (5.0)	62.1 (2.5)	1.1 (-2.1–4.4)	0.55 (-2.6–3.8)	0.63 (-4.2–5.5)
≠ intragroups	3.0 (0.9–5.0)^a^	2.8 (0.4–5.1)^a^	0.64 (-0.1–1.4)			
Social aspects						
Pre	75.1 (2.8)	76.0 (2.8)	76.2 (3.8)	- 1.1 (-4.2–2.0)	-0.21 (-3.2–2.7)	-0.88 (-3.6–1.8)
Post	76.5 (4.4)	79.6 (3.7)	76.5 (4.4)	0.5 (-2.7–3.7)	3.1 (-0.47–6.6)	-2.6 (-5.5–0.35)
≠ intragroups	1.8 (0.4–3.3)^a^	3.6 (1.0–6.1)^a.b^	0.28 (-0.9–1.4)			
Emotional aspects						
Pre	74.3 (5.2)	75.1 (5.1)	72.2 (4.1)	2.1 (-1.9–6.1)	2.8 (-1.0–6.8)	–0.76 (-5.8–4.2)
Post	76.1 (5.6)	76.0 (4.9)	72.3 (4.6)	3.7 (-0.7–8.2)	3.6 (-0.46–7.7)	0.11 (–5.0–5.2)
≠ intragroups	1.7 (0.2–3.3)^a^	0.9 (-1.1–2.9)	0.14 (-0.5–0.8)			
Mental health						
Pre	71.2 (3.9)	73.6 (6.1)	70.5 (5.4)	0.72 (-3.6–5.1)	3.1 (-1.8–8.0)	-2.3 (-7.4–2.6)
Post	73.3 (2.7)	74.1 (6.9)	70.8 (5.4)	2.47 (-1.5–6.5)	3.2 (-1.9–8.4)	-0.7 (-6.0–4.4)
≠ intragroups	2.11 (0.2–3.9)^a^	0.5 (-2.5–3.5)	0.35 (-2.0–2.7)			

The data regarding the domains of quality of life are presented by the mean (standard deviation) and the intra and intergroup differences by the mean difference (confidence interval of 95%).

^a^ Significant difference (95%CI does not go through zero).

^b^ Tukey *post hoc* test (unidirectional ANOVA), p < 0.05 in relation to the CG.

^c^ Tukey *post hoc* test (unidirectional ANOVA), p < 0.05 compared with the VR group.

The intra-group analysis of the results of the SF36 reveals that the individuals who underwent VR treatment showed a significant improvement in the quality of life in the domains limitation by physical aspects, general health status, vitality and social aspects. The individuals who underwent the LG integrative complementary treatment obtained statistically significant improvement in all domains, including, besides the aforementioned, functional capacity, pain, emotional aspects and mental health. The individuals in CG did not differ in the two assessments in any domain.

It is possible to perceive by ANOVA followed by Tukey *post hoc* test that the interventions caused different variations between the groups. The variation caused by the LG integrative complementary intervention was higher than the variation observed in the CG for the domains functional capacity, limitation by physical aspects and general health status, and also higher than the variation found after the VR intervention in the domain pain. The variation caused by the VR intervention was higher than the variation observed in the CG in the domains limitation by physical aspects and social aspects.

**Figure 2:**
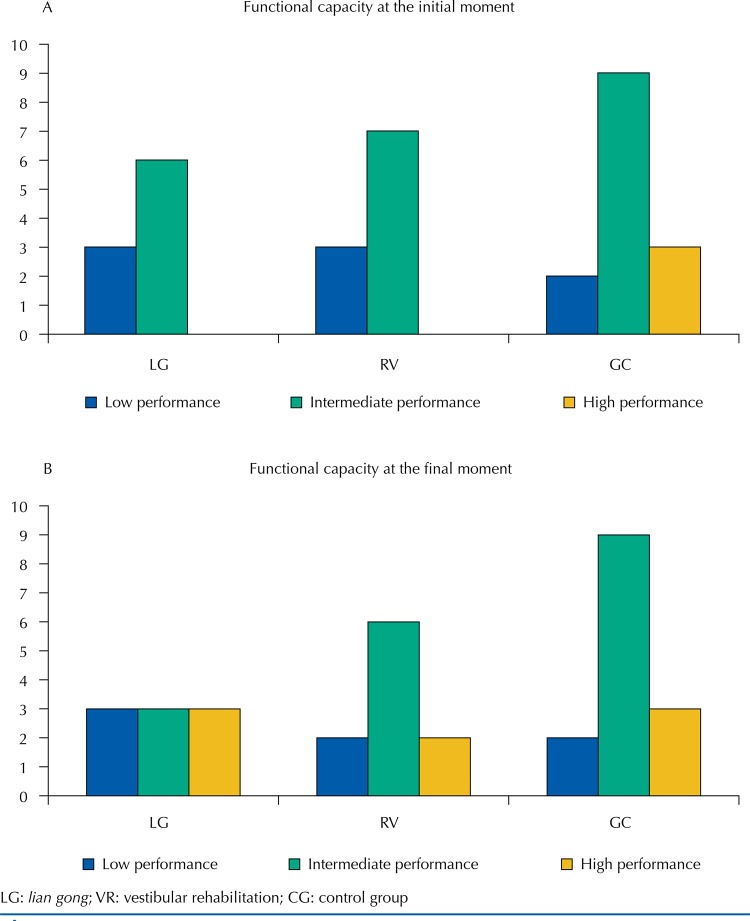
Distribution of functional classifications before and after interventions.

LG: *lian gong*; VR: vestibular rehabilitation; CG: control group

In the intergroup comparison, we verified a significant difference between the variations caused by the interventions between the LG and CG groups in the general health domain. In other words, the integrative complementary treatment improved the quality of life in relation to the general health aspect when compared with the CG. Figure 2 shows the results of the SPPB at the initial and final moments. No difference was found between the groups at the initial (p = 0.27) and final (p = 0.66) moments.

## DISCUSSION

In this clinical trial, the effects of LG after 12 weeks of intervention were investigated, and the results elucidate positive implications of LG on the quality of life of individuals with dizziness in the functional capacity aspects, limitation by physical aspects and general health status. The LG and VR interventions showed similar results, except in the domain pain, in which the LG obtained better results. Such findings indicate the positive effects of a complementary integrative practice advocated by the Ministry of Health^3^ in improving the quality of life of patients with dizziness, one of the main complaints of the patient in PHC^5^.

The literature is incisive when proposes the benefits of oculomotor activities, of body and cephalic rotation, of static and dynamic balance as promoters of habituation and vestibular compensation in patients with dizziness^4,8,9,17,21^. From this perspective, the hypothesis of this study is that the LG is beneficial to patients with dizziness arising from non-central causes, positively impacting the functional capacity and quality of life of patients with dizziness In the PHC. The fact that the variations in the quality of life scores did not differ between the LG and the VR groups, except for the domain pain, with more expressive results in the LG group, indicates the real possibility of the patient with dizziness, duly indicated by the physician of the PHC, to participate in the LG as an alternative effective treatment to improve the quality of life. These findings are the base of the functions of solvability and accountability of PHC, as well as the principles of accessibility, integrality, continuity, bonding and humanization^22^. PHC and especially the FHS, with its longitudinality, territorial and cultural immersion, facilitate the exploitation of therapeutic bonds and the use of community resources of various types, constituting a strong and important affinity between the integrative complementary practices and health care^22^. Given this context, LG enhances a strong partnership in the care of the individual with dizziness.

Table 1 shows that the participants of this research did not differ in the socio-demographic aspects, body function and structure, limitations by activities and other health conditions. The sample was mainly composed of women, older women, with visual alterations and reports of anxiety, common symptoms in the individual with dizziness^23^. Moreover, limitations involving locomotion were reported by a large part of the participants, as well as the presence of hypertension, morbidity also associated with functional decline, higher risk of falls and dizziness^24^. The similarity and homogeneity of the groups in the initial evaluation were confirmed by the statistical analyses of the outcomes quality of life and functional capacity, which did not differ between the groups.

The analysis of the results in the context of PHC requires a differentiated approach of the domains general health, functional capacity and limitation by physical aspects, which significantly improved in the individuals participating in the LG. This can be explained because this technique aims to deal with the subjects in their integrality, that is, in addition to stimulating ocular fixation, postural stability and neuronal plasticity as the conventional VR, it has proven benefits in reducing osteomuscular pain, in the gain in range of motion, muscular strength and flexibility, as well as in the reduction in stress and psychosomatic consequences caused by diseases^12,18^.

In clinical trials in PHC with individuals with dizziness, the method of vestibular rehabilitation intervention proved to be effective in reducing the impact of dizziness on the quality of life of the participants, which were evaluated by the Dizziness Handcap Inventory before and after intervention^8,9,17^. In this study, the LG and VR interventions did not differ in relation to the general health, functional capacity, vitality, mental health, emotional and social aspects, i.e., the LG produces effects similar to vestibular rehabilitation in the quality of life of the participants. These findings indicate the advancement in the integral care of the subject, improving the care of patients with dizziness, who have in the PHC their first contact.

When analyzing the results of SPPB, no statistically significant difference was found between the groups at the initial and final moments. However, a change in the performance of the individuals submitted to the interventions was observed (Figure 2). In the LG *g*roup, 33% of the individuals acquired high performance at the end of treatment, as well as 20% in the VR group. Such findings can be justified by the literature, which describes better results of SPPB in active older adults compared with the sedentary ones^25^ and improvement in the functional capacity of older individuals undergoing physical activity^26^. The functional capacity of the older adults is known to be associated with their quality of life^27^ and can be improved through interventions performed in primary health care. Thus, we believe that LG, an integrative and complementary practice advocated by the Ministry of Health^3^, is a relevant strategy to optimize such aspects and reduce the risk of falls in older adults, minimizing negative health outcomes, including the restriction of activities and the decline in functional and physical performance.

No clinical trials were found in the literature to verify the effectiveness of LG as a therapeutic strategy to reduce dizziness symptoms. Among the integrative and complementary practices, there are records of the effects of Tai Chi on the improvement of balance^10^, of yoga in postural control^28^ and of acupuncture in the improvement of vestibular migraine^29^, in addition to ongoing research to verify the effect of acupuncture on chronic dizziness^30^.

This study, of unprecedented character, provides scientific evidence of the benefits of LG in the care of individuals with dizziness in PHC. Studies evaluating the integrative complementary practices are essential in the national and international contexts, since they contribute significantly to the health care of the world population^1^ and to the construction of care networks focused on the subjects in their social or family contexts. Moreover, they value knowledge or non-biomedical practices, experiences and care techniques, stimulus to self-healing, active participation and empowerment of patients^22^.

## CONCLUSION

Based on the results presented, LG improves the quality of life of individuals with dizziness, without altering the functional capacity. The improvement observed covered more domains of quality of life than a traditional treatment modality, the VR. Therefore, it is a useful rehabilitation strategy in PHC for the treatment of people with dizziness. However, the effects of LG on long-term clinical and functional parameters still need to be investigated.
