# Supplementary material for: Impact of lian gong on the quality of life of individuals with dizziness in primary care
Source: Rev Saude Publica. 2019 Sep 12;53:73. doi: 10.11606/s1518-8787.2019053001234 (PMC6752643; doi:10.11606/s1518-8787.2019053001234)
Supplement: Translation

# Translation: Impacto do *lian gong* na qualidade de vida de indivíduos com tontura na atenção primária

**Authors:** Aline Lamas Lopes, Stela Maris Aguiar Lemos, Pedro Henrique Scheidt Figueiredo, Juliana Nunes Santos

**Affiliations:** I Universidade Federal de Minas Gerais, Faculdade de Medicina, Programa de Pós-Graduação em Ciências Fonoaudiológicas. Belo Horizonte, MG, Brasil; II Universidade Federal de Minas Gerais, Faculdade de Medicina, Departamento de Fonoaudiologia. Belo Horizonte, MG, Brasil; III Universidade Federal dos Vales do Jequitinhonha e Mucuri (UFVJM), Faculdade de Ciências Biológicas e da Saúde, Departamento de Fisioterapia. Diamantina, MG, Brasil

**Keywords:** Tontura, reabilitação, Terapias Complementares, Atenção Primária à Saúde, Ensaio Clínico Controlado Aleatório

## Abstract

**OBJETIVO:**

Avaliar os efeitos da prática do *lian gong* como estratégia de reabilitação na atenção primária à saúde sobre a qualidade de vida e capacidade funcional de pessoas com tontura.

**MÉTODOS:**

Trata-se de ensaio clínico randomizado-controlado. Participaram 36 voluntários, com queixa de tontura ou vertigem sem a presença de sinais centrais, encaminhados pelo médico da atenção primária à saúde. Os indivíduos foram aleatoriamente alocados para as três condições experimentais: grupo *lian gong* (n = 11), grupo reabilitação vestibular (n = 11) e grupo controle (n = 14). As intervenções foram semanais, em grupo, com duração de 12 sessões. Os participantes foram avaliados antes e após a intervenção quanto à qualidade de vida pelo *36-Item Short Form Health Survey* e quanto à capacidade funcional pelo *Short Physical Performance Battery*.

**RESULTADOS:**

Observou-se aumento dos scores de todos os domínios do *Short Form Health Survey* após intervenção no grupo *lian gong*. Essa variação foi maior que a observada no grupo controle para os domínios capacidade funcional, limitação por aspectos físicos e estado geral de saúde, e também superior à encontrada após a intervenção grupo reabilitação vestibular no domínio dor. Não houveram diferenças no *Short Physical Performance Battery*.

**CONCLUSÕES:**

Com base nos resultados apresentados, o *lian gong* melhora a qualidade de vida de indivíduos com tontura, sem alterar a capacidade funcional.

## INTRODUÇÃO

Pesquisas revelam o uso crescente da medicina complementar e alternativa (MCA) em todo o mundo como forma de melhorar a saúde e o bem-estar, assim como aliviar sintomas associados a doenças crônicas ou efeitos colaterais dos tratamentos convencionais^1,2^. O interesse pelo uso da MCA aumentou principalmente em países desenvolvidos como França, Canadá, Alemanha e Itália, nos quais 70% a 90% da população fazem uso de seus recursos e práticas terapêuticas^1^. Nos Estados Unidos, observou-se em 2012 que 33% dos adultos utilizaram alguma prática complementar de saúde^2^.

No Brasil, as terapias complementares são denominadas práticas integrativas e complementares (PIC). Foram instituídas pela portaria 971/2006 do Ministério da Saúde^3^ e correspondem a um conjunto de terapêuticas que incluem exercícios físicos orientais como *lian gong* (LG), tai chi chuan, além da acupuntura, auriculoterapia, homeopatia, termalismo, fitoterapia e massagem oriental. A utilização das terapias alternativas e complementares está em ascensão, assim como sua aceitação no controle e tratamento de várias condições crônicas de saúde como HIV/Aids, hipertensão, colesterol alto, insônia, bronquite, diabetes, câncer^1^, tonturas e vertigens^4^, entre outros.

Entre as razões mais comuns para buscar uma consulta médica na atenção primária à saúde (APS), está a tontura. Ela é considerada o sintoma mais frequente em todo o mundo, ocorrendo em todas as faixas etárias, principalmente em adultos e idosos^5^, sendo multifatorial na maioria dos casos, de origem vestibular e não vestibular^6^. Entre as causas de origem vestibular destacam-se a vertigem posicional paroxística benigna, presbivertigem, doença de Ménière, neurite vestibular, insuficiência vertebrobasilar, migrânea vestibular e vestibulopatias secundárias às infecções labirínticas^6^. Já as causas não vestibulares diversas são as doenças que alteram diretamente essas funções, predominando as cardiovasculares e ortopédicas e a diminuição da acuidade visual^6,7^.

Acredita-se que a tontura e a vertigem, como sintomas de afecções vestibulares, estejam presentes em 5% a 10% da população mundial, sendo o sintoma mais comum após os 65 anos^6,7^. Estima-se que um terço dos adultos mais velhos tenham sintomas de tontura no período de um ano^6^.

A literatura apresenta como formas de tratamento da tontura e da vertigem o uso de medicação, recursos cirúrgicos, a reabilitação vestibular (RV) tradicional e a RV com a utilização de inovações tecnológicas e de realidades virtuais, além das práticas alternativas complementares^4^. Há evidências de efeitos positivos da RV na APS, com melhoras no controle postural, capacidade funcional e qualidade de vida dos usuários, sendo essa opção terapêutica a mais abordada na reabilitação dos indivíduos^8,9^. Dentre as práticas alternativas, as terapias de acupuntura e tai chi têm apresentado resultados favoráveis, indicando melhora do equilíbrio e da qualidade do sono, assim como redução do risco de quedas em indivíduos com tontura^4,10,11^.

No Brasil, o LG tem sido realizado na APS como parte da Política Nacional de Práticas Integrativas e Complementares no SUS^3^. O LG é uma ginástica terapêutica composta por exercícios baseados no conhecimento e vivência das artes corporais e marciais chinesas. Ele estimula a persistência de treinar e exercitar o corpo por intermédio de movimentos firmes e suaves que minimizam e eliminam as tensões musculares, alongam ligamentos e tendões, corrigem a postura física, estimulam a percepção e integração dos sentidos e otimizam a coordenação motora, o equilíbrio e a consciência corporal, além de promover a harmonização entre corpo e mente, reduzindo os sintomas de ansiedade e depressão^12,13^.

O sistema completo do LG é composto por três partes, totalizando 54 exercícios. A primeira parte (anterior) compreende 18 movimentos, a fim de prevenir e tratar dores no pescoço, ombros, costas, região lombar, glúteos e pernas. Já a segunda parte (posterior), também composta por 18 exercícios, destina-se à prevenção e tratamento de dores nas articulações, tenossinovites e disfunções dos órgãos internos. A terceira parte, denominada *i qi gong*, contempla um conjunto de 18 exercícios que tem como finalidade a prevenção e o tratamento da bronquite crônica e da debilidade funcional do coração e pulmões, assim como de outras doenças crônicas das vias respiratórias. A realização dos exercícios é acompanhada de música tocada por instrumentos chineses, cujo arranjo estabelece o ritmo apropriado para a execução dos movimentos. Assim, cada parte de 18 exercícios leva aproximadamente 12 minutos para ser realizada^12,13^.

Em todas as partes, há realização de movimentos de rotação cefálica associados aos movimentos oculares de perseguição e fixação visual, simultâneos ou não a movimentos corporais mais amplos, os quais estimulam os sistemas vestíbulo-ocular e vestíbulo-espinhal, essenciais no processo de reabilitação vestibular^4^. Tanto na parte anterior quanto na parte posterior do LG, há movimentação cefálica lateral ou ântero-superior, e os movimentos de fixação e perseguição ocular podem ser observados em 100% dos exercícios. Já a prática do *i qi gong*, além de exigir movimentação cefálica e movimentos de perseguição e fixação ocular, exercita o equilíbrio estático e dinâmico dos indivíduos por meio de exercícios corporais alternando olhos abertos e fechados^12,13^. Assim como na RV, acredita-se que a repetição dos exercícios praticados no LG promova a estabilização visual e aumentem a interação vestíbulo-visual durante a movimentação da cabeça, proporcionando melhor estabilidade estática e dinâmica nas situações de conflito sensorial, o que potencializa a os mecanismos de adaptação vestibular^4,6,14^.

Não há estudos que investiguem os efeitos dessa prática integrativa complementar nos indivíduos com tontura. No entanto, acredita-se que o LG, por suas características peculiares, seja benéfico a essa população e reduza o impacto da tontura na qualidade de vida dos participantes. Há de se considerar ainda, conforme preconizado pela Organização Mundial de Saúde, a necessidade emergente de pesquisas científicas para avaliar a qualidade, segurança e efetividade das práticas da MCA^1^ em todo o mundo.

Assim sendo, o objetivo do presente estudo é avaliar os efeitos da prática do LG como estratégia de reabilitação na APS sobre a qualidade de vida e capacidade funcional de pessoas com tontura.

## MÉTODOS

### Delineamento

Trata-se de ensaio clínico randomizado controlado, de alocação paralela, com dois braços. O projeto intitulado “A Viabilidade e Efetividade do Programa de Reabilitação Vestibular na Atenção Primária à Saúde” foi aprovado pelo Comitê de Ética em Pesquisa institucional da Universidade Federal de Minas Gerais sob o número CAAE 15987713.5.00005149 e pelo Registro Brasileiro de Ensaios Clínicos sob o código RBR-2nxt6y. Todos os participantes assinaram o termo de consentimento livre e esclarecido.

Os gerentes das unidades de saúde participantes foram informados sobre a realização do estudo e assinaram a carta de anuência. Os médicos da APS que voluntariamente aceitaram participar da pesquisa encaminharam os usuários com queixas de tontura ou vertigem sem presença de sinais e sintomas de alterações centrais por meio da reunião de matriciamento da equipe de saúde da família (ESF) com o núcleo de apoio em saúde da família (NASF).

### Cenário do Estudo

O presente estudo foi realizado em dois centros de saúde da rede SUS de uma metrópole brasileira.

### Amostra

No período de maio a dezembro de 2016, os indivíduos foram recrutados para a participação no estudo. Os critérios de inclusão considerados foram: idade igual ou superior a 18 anos, ser usuário do SUS e morador ou trabalhador das áreas de abrangência dos centros de saúde participantes da pesquisa, apresentar queixa de tontura ou vertigem com ausência de sinais ou sintomas centrais, indicação médica para participação nos grupos propostos e assinatura do termo de consentimento. Os critérios de exclusão foram: presença de deficiência intelectual ou física ou de transtornos mentais que impedissem a realização das atividades propostas nos grupos, desistência ou não adesão ao tratamento com mais de quatro faltas aos encontros, presença de gestação e avaliação fonoaudiológica compatível com quadro de vertigem posicional paroxística benigna (VPPB).

Para a determinação do número de sujeitos, foi utilizado o programa estatístico G*Power 3.1. O tamanho da amostra foi baseado no estudo de Yardley et al.^17^, com os resultados da comparação dos escores médios e desvio-padrão (DP) do *Dizziness Handicap Inventory* (DHI) entre dois grupos de pacientes participantes de um ensaio clínico randomizado para verificar a efetividade da reabilitação vestibular na APS. Para o presente ensaio foram necessários nove usuários para cada grupo, considerando poder (erro tipo beta, tipo I) de 95%, alfa igual a 0,05 e tamanho do efeito de 1,95. Houve um acréscimo de perdas de 20% (n = 5), totalizando 32 sujeitos.

Os sujeitos da pesquisa foram encaminhados pelo médico da APS e avaliados por um pesquisador velado para os grupos. Após a análise dos critérios de inclusão e exclusão, os sujeitos foram alocados aleatoriamente em três grupos: grupo LG, de método complementar/integrativo; grupo RV, de método convencional, e grupo controle (GC). A randomização foi realizada por meio de um sorteio simples. Tiras de papel contendo a indicação de um dos três grupos foram alocadas em envelopes opacos. No momento da entrada do voluntário no estudo, um pesquisador sorteava o grupo na qual o voluntário seguiria.

Para caracterização da amostra, instrumentos elaborados pelas pesquisadoras foram usados para avaliar os aspectos sociodemográficos, o equilíbrio e as queixas, sinais e sintomas da tontura e vertigem dos voluntários. Para esses últimos, foram colhidas informações sobre a história clínica do usuário e sua relação com a queixa apresentada.

### Intervenções

A intervenção proposta para o grupo LG foi pautada no protocolo preconizado pelo autor da técnica, Dr. Zuang Yuan Ming, conforme modelo de aula já estabelecido pela Prefeitura de Belo Horizonte: série anterior, série posterior e *i qi gong*. Foram executados 54 exercícios, coordenados com a respiração, de forma lenta e contínua, que atuam no indivíduo como um todo. Além de tratar e prevenir dores osteomusculares, esses exercícios otimizam a função cardiorrespiratória, estimulam o domínio do equilíbrio, a consciência corporal, a estabilização postural e a fixação ocular^12,13,18^. Já no grupo RV, a intervenção foi baseada nos protocolos para reabilitação vestibular de Norré^14^, Cawthorne e Cooksey^15^ e Herdman^16^, com a seleção de exercícios para a estabilização postural, fixação ocular e treinamento para manutenção do equilíbrio. Os indivíduos do grupo controle não receberam tratamento.

As intervenções (RV e LG) foram ministradas por uma das pesquisadoras, fonoaudióloga do NASF. As sessões de 50 minutos foram realizadas semanalmente em caráter coletivo (grupos entre cinco e sete participantes). O início das intervenções foi em julho de 2016 e o término em abril de 2017, de modo que os usuários encaminhados foram inseridos nos grupos logo após a avaliação inicial e permaneceram até completar 12 sessões.

### Desfechos

As avaliações pré e pós-intervenção foram realizadas por uma fonoaudióloga que desconhecia a qual dos grupos o paciente avaliado pertencia. Os desfechos primários foram os resultados do questionário genérico de avaliação de qualidade de vida *36-Item Short Form Health Survey* (SF36)^19^. Já a capacidade funcional dos participantes, avaliada pelo *Short Physical Performance Battery* (SPPB)^20^, foi considerada como desfecho secundário.

O SF36 foi criado para ser um questionário genérico de avaliação em duas partes: a primeira para avaliar o estado de saúde (com questões relacionadas à mobilidade física, dor, sono, energia, isolamento social e reações emocionais) e a segunda parte para avaliar o impacto da doença na qualidade de vida diária do paciente. Trata-se de um questionário multidimensional formado por 36 itens, subdivididos em oito escalas ou componentes: capacidade funcional, aspectos físicos, dor, estado geral de saúde, vitalidade, aspectos sociais, aspectos emocionais e saúde mental. O SF36 foi analisado pelo escore final variando de zero a cem pontos (obtido por meio de cálculo de *raw scale*), na qual zero reflete o pior estado de saúde e cem, o melhor^19^.

O *Short Physical Performance Battery* (SPPB) foi adaptado para a língua portuguesa por Nakano^20^. É um teste amplamente utilizado na prática clínica para avaliação funcional de idosos. Ele compreende três etapas, cada uma delas com pontuação de até quatro pontos, totalizando o escore final de no máximo 12 pontos. Na primeira, o indivíduo permanece inicialmente em posição ortostática com os pés paralelos, em seguida, com os pés em semitandem e depois na postura tandem. Na segunda etapa, foi medida a velocidade de marcha em um percurso de quatro metros, dividindo-se a distância percorrida pelo tempo gasto. Na terceira, foi medido o tempo que o idoso leva para realizar o teste de assentar e levantar de uma cadeira sem braços por cinco vezes^20^. O escore total do SPPB foi a somatória dos resultados dos testes de equilíbrio, velocidade de marcha e força dos membros inferiores. Os indivíduos que obtiveram entre zero e seis pontos foram classificados como de baixa performance, entre sete e nove pontos, performance intermediária, e entre 10 e 12, alta performance.

### Análise Estatística

Foi realizada no *software* SPSS 19.0 (*Statistical Package for the Social Sciences*). O teste de Shapiro-Wilk foi empregado para análise da distribuição normal das variáveis contínuas e os dados estão apresentados em média e desvio-padrão. As variáveis de caracterização da amostra foram comparadas entre os grupos pela análise de variância (Anova) unidirecional (*one way*) ou teste de Kruskal-Wallis, conforme a prova de normalidade, com *post hoc* pelo teste de Tukey, se necessário. A comparação da distribuição das variáveis categóricas entre os grupos foi realizada pelo teste qui-quadrado e está apresentada em números absolutos e frequências relativas.

Os efeitos das intervenções nos domínios do SF36 foram comparados por meio dos intervalos de confiança de 95% (IC95%) das diferenças entre as médias nos momentos inicial e final, e também das diferenças intergrupos (LG *versus* RV; LG *versus* GC; RV *versus* GC) nos momentos inicial e final. A comparação entre as diferenças foi realizada pela Anova unidirecional com *post hoc* pelo teste de Tukey*.* Para a análise dos resultados do SPPB, foi utilizado o teste do qui-quadrado. O nível de significância adotado foi de 5%.

## RESULTADOS

O fluxo dos voluntários no estudo está apresentado na Figura 1. Inicialmente foram encaminhados 86 usuários para possível participação na pesquisa, porém 50 foram excluídos por apresentarem sinais clínicos compatíveis com VPPB durante a avaliação fonoaudiológica. Assim, 36 voluntários foram aleatoriamente alocados para as três condições experimentais, sendo 11 (31%) no grupo LG, 11 (31%) no grupo RV e 14 (38%) no GC. Após o início das intervenções, um indivíduo do grupo LG abandonou o tratamento devido a uma oportunidade de trabalho no mesmo horário e dois faltaram a mais de quatro sessões, um do grupo LG e um do grupo RV. Assim, foram analisados 33 usuários que concluíram o estudo, sendo 29 (87,9%) do sexo feminino, com idade média de 63 anos (DP = 5,17), mínima de 52 e máxima de 72 anos (Tabela 1).

**Figura 1:** Fluxograma de participação no ensaio clínico randomizado.

VPPB: vertigem posicional paroxística benigna

**Tabela 1:** Caracterização dos participantes do estudo.

Variável	Intervenção (n = 9)	Intervenção (n = 10)	Controle (n = 14)	p
		
	(LG)	(RV)	(GC)	
		
	Média e desvio-padrão/n e freq. relativa (%)	Média e desvio-padrão/n e freq. relativa (%)	Média e desvio-padrão/n e freq. relativa (%)	
Aspectos sociodemográficos				
Idade^a^ (anos)	64 (4,5)	64,7 (4,4)	61,1 (5,6)	0,20
Sexo feminino^b^	8 (88,8)	9 (90)	12 (85,7)	0,94
Função e estrutura do corpo				
Tontura^b^	9 (100)	10 (100)	14 (100)	1,00
Vertigem^b^	9 (100)	10 (100)	14 (100)	1,00
Ansiedade^b^	9 (100)	8 (80)	11 (78,5)	0,33
Zumbido^b^	6 (66,6)	7 (70)	11 (78,5)	0,80
Sensação de cabeça oca^b^	7 (77,7)	5 (50)	6 (42,9)	0,24
Sensação de visão borrada^b^	6 (66,5)	4 (40)	6 (42,9)	0,43
Alterações visuais^b^	7 (77,7)	9 (80)	12 (85,7)	0,75
Limitação de atividades				
Tropeçar	3 (33,3)	4 (40)	9 (64,3)	0,28
Cambalear	5 (55,5)	9 (90)	12 (85,7)	0,13
Virar o pé	4 (44,4)	3 (30)	10 (71,4)	0,12
Cair	3 (33,3)	5 (50)	10 (71,4)	0,19
Não fazer atividade física	5 (55,5)	6 (60)	10 (71,4)	0,71
Outras condições de saúde				
HAS	5 (55,5)	5 (50)	4 (28,6)	0,37
Diabetes	3 (33,3)	1 (10)	3 (21,4)	0,46
Depressão	3 (33,3)	3 (30)	2 (14,3)	0,51
Enxaqueca	2 (22,2)	5 (50)	2 (14,3)	0,14

LG: *lian gong*; RV: reabilitação vesibular; GC: grupo controle; Freq.: frequência; HAS: hipertensão arterial sistêmica

^a^ Anova

^b^ Teste qui-quadrado.

Na Tabela 1 também podem ser observadas as características dos usuários dos grupos LG, RV e GC. Os grupos não diferiram entre si em relação às características demográficas, função e estrutura do corpo, limitação de atividades e outras condições de saúde (p > 0,05). Na Tabela 2 são apresentadas as análises intragrupo e intergrupos da qualidade de vida dos usuários nos momentos inicial e final.

**Tabela 2:** Efeitos das intervenções sobre a qualidade de vida.

Domínios do SF36	Grupos			Diferença intergrupos		
	
	*Lian gong* (LG)	Reabilitação vestibular (RV)	Controle (GC)	LG *versus* GC	RV *versus* GC	LG *versus* RV
Estado geral de saúde						
Pré	81,4 (3,2)	81,7 (4,6)	80,5 (1,7)	0,94 (-1,1–3,0)	1, 2 (-1,5–3,9)	0,25 (4,2–3,6)
Pós	84,8 (4,1)	83,1 (4,8)	80,5 (1,5)	4,3 (1,7–6,8)^a^	2,5 (-0,34–5,4)	1,7 (-2,5–6,1)
≠ intragrupos	3,44 (0,7–6,1)^a,b,c^	1,4 (0,01–2,7)^a^	0,71 (-0,5–0,6)			
Capacidade funcional						
Pré	69,7 (7,3)	67,4 (6,3)	71,8 (7,1)	-2,0 (-8,5–4,3)	- 4,4 (-10,3–2,8)	2,3 (-4,2–8,9)
Pós	72,8 (7,1)	69,2 (6,3)	71,6 (6,9)	0,91 (-5,3–7,1)	-2,4 (-8,1–3,3)	3,3 (-3,3–9,8)
≠ intragrupos	2,77 (1,2–4,3)^a,b^	1,8 (-0,5–4,1)	-0,21 (-1,3–0,8)			
Limitação por aspectos físicos						
Pré	82,7 (4,2)	80,9 (4,2)	81, 0 (4,4)	1,8 (-2,1–5,7)	-0,1 (-3,8–3,6)	1,8 (-2,2–6,0)
Pós	84,3 (4,5)	82,4 (5,2)	80,5 (4,8)	2,0 (-0,47–7,9)	1,8 (-2,5–6,1)	1,9 (-2,8–6,7)
≠ intragrupos	1,5 (0,3–2,7)^a,b^	1,5 (0,2–2,7)^a,b^	-0,42 (-1,4–0,5)			
Dor						
Pré	78,4 (5,3)	79,0 (5,8)	81,2 (3,0)	-2,8 (-6,4–0,8)	-2,2 (-6,0–1,4)	-0,55 (-5,9–4,8)
Pós	8,18 (4,8)	77,3 (8,3)	81,2 (2,9)	0,67 (-2,6–4,0)	-3,9 (-8,8–1,0)	4,5 (-2,1–11,2)
≠ intragrupos	3,4 (0,8–6,0)^a,c^	-1,7 (-6,7–3,3)	-0,07 (-0,9–0,7)			
Vitalidade						
Pré	60,3 (4,1)	59,9 (3,2)	61,5 (2,8)	1,4 (-4,1–1,8)	-1,6 (-4,1–0,97)	0,43 (-3,1–4,0)
Pós	63,3 (5,0)	62,7 (5,0)	62,1 (2,5)	1,1 (-2,1–4,4)	0,55 (-2,6–3,8)	0,63 (-4,2–5,5)
≠ intragrupos	3,0 (0,9–5,0)^a^	2,8 (0,4–5,1)^a^	0,64 (-0,1–1,4)			
Aspectos sociais						
Pré	75,1 (2,8)	76,0 (2,8)	76,2 (3,8)	- 1,1 (-4,2–2,0)	-0,21 (-3,2–2,7)	-0,88 (-3,6–1,8)
Pós	76,5 (4,4)	79,6 (3,7)	76,5 (4,4)	0,5 (-2,7–3,7)	3,1 (-0,47–6,6)	-2,6 (-5,5–0,35)
≠ intragrupos	1,8 (0,4–3,3)^a^	3,6 (1,0–6,1)^a,b^	0,28 (-0,9–1,4)			
Aspectos emocionais						
Pré	74,3 (5,2)	75,1 (5,1)	72,2 (4,1)	2,1 (-1,9–6,1)	2,8 (-1,0–6,8)	–0,76 (-5,8–4,2)
Pós	76,1 (5,6)	76,0 (4,9)	72,3 (4,6)	3,7 (-0,7–8,2)	3,6 (-0,46–7,7)	0,11 (–5,0–5,2)
≠ intragrupos	1,7 (0,2–3,3)^a^	0,9 (-1,1–2,9)	0,14 (-0,5–0,8)			
Saúde mental						
Pré	71,2 (3,9)	73,6 (6,1)	70,5 (5,4)	0,72 (-3,6–5,1)	3,1 (-1,8–8,0)	-2,3 (-7,4–2,6)
Pós	73,3 (2,7)	74,1 (6,9)	70,8 (5,4)	2,47 (-1,5–6,5)	3,2 (-1,9–8,4)	-0,7 (-6,0–4,4)
≠ intragrupos	2,11 (0,2–3,9)^a^	0,5 (-2,5–3,5)	0,35 (-2,0–2,7)			

Os dados referentes aos domínios da qualidade de vida estão apresentados pela média (desvio-padrão) e as diferenças intra e intergrupos pela média da diferença (intervalo de confiança a 95%).

^a^ Diferença significativa (IC95% não passa pelo zero).

^b^*Post hoc* pelo Tukey test (Anova unidirecional), p < 0,05 em relação ao GC.

^c^ *Post hoc* pelo Tukey test (Anova unidirecional), p < 0,05 em relação ao grupo RV.

A análise intragrupo dos resultados do SF36 revela que os indivíduos que realizaram o tratamento de RV apresentaram melhora significativa da qualidade de vida nos domínios limitação por aspectos físicos, estado geral de saúde, vitalidade e aspectos sociais. Os indivíduos que realizaram o tratamento complementar integrativo LG obtiveram melhora estatisticamente significante em todos os domínios, incluindo, além dos supracitados, capacidade funcional, dor, aspectos emocionais e saúde mental. Os indivíduos do GC não diferiram nas duas avaliações em nenhum domínio.

É possível perceber pela Anova seguida do *post hoc* de Tukey que as intervenções causaram variações distintas entre os grupos. A variação causada pela intervenção complementar integrativa LG foi maior do que a variação observada no GC para os domínios capacidade funcional, limitação por aspectos físicos e estado geral de saúde, e também superior à variação encontrada pós intervenção RV no domínio dor. Já a variação causada pela intervenção RV foi maior do que a variação observada no GC nos domínios limitação por aspectos físicos e aspectos sociais.

Na comparação intergrupos foi possível verificar diferença significativa entre as variações causadas pelas intervenções entre o grupo LG e GC no domínio estado geral de saúde. Ou seja, a realização do tratamento complementar integrativo foi efetiva na melhora da qualidade de vida em relação ao aspecto geral de saúde quando comparado ao GC. Na Figura 2 podem ser visualizados os resultados do SPPB nos momentos inicial e final. Não houve diferença entre os grupos nos momentos inicial (p = 0,27) e final (p = 0,66).

**Figura 2:** Distribuição das classificações funcionais antes e após as intervenções.

LG: *lian gong*; RV: reabilitação vestibular; GC: grupo controle

## DISCUSSÃO

No presente ensaio clínico, investigaram-se os efeitos do LG após 12 semanas de intervenção, e os resultados elucidam implicações positivas do LG na qualidade da vida de indivíduos com tontura nos aspectos capacidade funcional, limitação por aspectos físicos e estado geral de saúde. As intervenções LG e RV apresentaram resultados similares, exceto no domínio dor, no qual o LG obteve melhores resultados. Tais achados apontam para os efeitos positivos de uma prática integrativa complementar preconizada pelo Ministério da Saúde^3^ na melhoria da qualidade de vida de usuários com tontura, uma das principais queixas do usuário na APS^5^.

A literatura é incisiva ao propor os benefícios de atividades oculomotoras, de rotação corporal e cefálica, de equilíbrio estático e dinâmico como favorecedores da habituação e compensação vestibular em pacientes com tontura^4,8,9,17,21^. Nessa perspectiva, a hipótese do presente estudo é que o *LG* seja benéfico aos usuários com tonturas advindas de causas não centrais, impactando positivamente a capacidade funcional e qualidade de vida dos usuários com tontura na APS. O fato de as variações dos escores de qualidade de vida não diferirem entre o grupo LG e o RV, exceto para o domínio dor, com resultados mais expressivos no grupo LG, aponta para a real possibilidade do usuário com tontura, devidamente indicado pelo médico da APS, participar do LG como alternativa de tratamento efetivo para a melhoria da qualidade de vida. Tais achados alicerçam as funções de resolubilidade e responsabilização da APS, assim como os princípios de acessibilidade, integralidade, continuidade, vínculo e humanização^22^. A APS e especialmente a ESF, com sua longitudinalidade, imersão territorial e cultural, facilitam a exploração dos vínculos terapêuticos e o uso de recursos comunitários de vários tipos, constituindo uma afinidade forte e importante entre as práticas complementares integrativas e a atenção à saúde^22^. Nesse contexto o LG potencializa uma forte parceria no cuidado do indivíduo com tontura.

Os dados apesentados na Tabela 1 revelam que os participantes deste ensaio não diferiram quantos aos aspectos sociodemográficos, função e estrutura do corpo, limitações por atividades e outras condições de saúde. A amostra foi majoritariamente composta por mulheres, idosas, com alterações visuais e relato de ansiedade, sintomas comuns no indivíduo com tontura^23^. Além disso, limitações envolvendo a locomoção foram referidas por grande parte dos participantes, assim como a presença de hipertensão, morbidade também associada ao declínio funcional, maior risco de quedas e tontura^24^. A semelhança e homogeneidade dos grupos na avaliação inicial foram confirmadas pelas análises estatísticas dos desfechos qualidade de vida e capacidade funcional, os quais não diferiram entre os grupos.

A análise dos resultados no contexto da APS exige uma abordagem diferenciada dos domínios estado geral de saúde, capacidade funcional e limitação por aspectos físicos, os quais melhoraram de forma significativa nos indivíduos participantes do LG. Isso pode ser atribuído ao fato de essa técnica se propor a trabalhar o sujeito em sua integralidade, ou seja, além de estimular a fixação ocular, a estabilidade postural e a plasticidade neuronal como a RV convencional, tem benefícios comprovados na redução das dores osteomusculares, no ganho da amplitude de movimento, força muscular e flexibilidade, bem como na redução do estresse e de consequências psicossomáticas causadas pelas doenças^12,18^.

Em ensaios clínicos na APS com indivíduos com tontura, o método de intervenção reabilitação vestibular mostrou-se efetivo para reduzir o impacto da tontura na qualidade de vida dos participantes, os quais foram avaliados pelo *Dizziness Handcap Inventory* antes e após a intervenção^8,9,17^. No presente estudo, as intervenções LG e RV não diferiram quanto aos domínios estado geral de saúde, capacidade funcional, vitalidade, saúde mental, aspectos emocionais e sociais, ou seja, o *LG* produz efeitos semelhantes à reabilitação vestibular na qualidade de vida dos participantes. Tais achados apontam para o avanço no cuidado integral do sujeito, aprimorando a assistência do usuário com tontura, que tem na APS o seu primeiro contato.

Ao analisar os resultados do SPPB, não se observou diferença estatisticamente significante entre os grupos nos momentos inicial e final. No entanto, percebe-se uma mudança na performance dos indivíduos submetidos às intervenções (Figura 2). No grupo *LG*, 33% dos indivíduos adquiriram alta performance ao término do tratamento, assim como 20% no grupo RV. Esses achados podem ser justificados pela literatura, que descreve melhores resultados do SPPB em idosos ativos em relação aos sedentários^25^ e melhora na capacidade funcional de idosos submetidos à atividade física^26^. Sabe-se que a capacidade funcional dos idosos tem associação com sua qualidade de vida^27^ e pode ser melhorada por meio de intervenções realizadas na atenção primária à saúde. Assim, acredita-se que o LG, prática integrativa e complementar preconizada pelo Ministério da saúde^3^, seja uma estratégia relevante para otimizar tais aspectos e ainda reduzir o risco de quedas em idosos, minimizando desfechos de saúde negativos, incluindo a restrição de atividades e o declínio do desempenho funcional e físico.

Não foram encontrados na literatura ensaios clínicos para verificar a efetividade do LG como estratégia terapêutica na redução dos sintomas da tontura. Dentre as práticas integrativas e complementares, há registros dos efeitos do tai chi na melhora do equilíbrio^10^, do yoga no controle postural^28^ e da acupuntura na melhora da migrânea vestibular^29^, além de pesquisas em andamento para verificar o efeito da acupuntura nas tonturas crônicas^30^.

O presente estudo, de caráter inédito, fornece evidências científicas dos benefícios do LG no cuidado de indivíduos com tontura na APS. Pesquisas de avaliação das práticas complementares integrativas são essenciais no contexto nacional e internacional, já que elas contribuem significativamente para o cuidado da saúde da população mundial^1^ e para a construção de redes de cuidados centradas nos sujeitos em seus contextos sociais ou familiares. Ademais, valorizam saberes ou práticas não biomédicas, vivências e técnicas de cuidado, estímulo à autocura, participação ativa e empoderamento dos usuários^22^.

## CONCLUSÃO

Com base nos resultados apresentados, o LG melhora a qualidade de vida de indivíduos com tontura, sem alterar a capacidade funcional. A melhora observada abrangeu mais domínios da qualidade de vida que uma modalidade de tratamento tradicional, a RV. Portanto, trata-se de uma estratégia de reabilitação útil na APS para o tratamento de pessoas com tontura. No entanto, os efeitos do LG sobre parâmetros clínicos e funcionais em longo prazo ainda precisam ser investigados.
